# Pyorrhoea Alveolaris with Practical Cases

**Published:** 1887-12

**Authors:** J. L. Gish

**Affiliations:** Jackson, Mich.


### THE DENTAL REGISTER.

VOL. XLI.] DECEMBER, 1887. [No. 12.

### COMMUNICATIONS.

### Pyorrhoea Alveolaris with Practical Cases.

BY J. L. GISH, M.D., D.D.S., JACKSON, MICH.

(Read before the Detroit Dental Society.)

*Gentlemen:* I ask your indulgence while we consider the very important disease commonly known as Pyorrhœa Alveolaris with a few practical cases.

*Definition:* Pyorrhœa Alveolaris is purely a descriptive name, or term, designating the place of lesion; namely, a flow of pus from the alveolar process and its associated parts.

*Causes:* Pyorrhœa Alveolaris may be hereditary or acquired. When hereditary, it is due to a want of tone, or power of resistance in those tissues, that later on become the seat of disease; but it is more commonly acquired by some *mechanical, chemical,* or *thermal* irritant, thus producing the simple or non-specific form of inflammation. In heredity the form of inflammation is specific. This disease is about equally divided between man and woman and comes under our notice, more generally, about middle life. This disease may be the result of catarrhal troubles of the throat and head.

*Pathological Anatomy:* The changes that take place in the alveolar process and its surrounding tissues depend entirely upon the kind and intensity of the degree of the inflammation. We will find the tissues in the pathological state, from the simple active and passive congestion with its serous effusions in the tissues to the absolute destruction of all involved structures.

In this disease, as in other manifestations of inflammation, we must note *first* the changes in the circulation ; and *second*, the changes that take place in the adjacent tissues. If we had the tissues involved, under the microscope, we would first notice a dilation of the capillary vessels, thus increasing the flow of circulatiou through the parts, but it is soon retarded, and the red corpuscles crowd together in masses, forming rouleau, while the white corpuscles, known as *leucocytes*, increase in number and move very slowly along the walls of the vessels. At this point we have the first stage of inflammation, namely, congestion.

Presently the circulation through the part or parts becomes completely closed, and stagnation is the result : but, while the stream of blood is being slowed in its course, the red corpuscles accumulate in the arteries and the white bodies find their place in the veins, whose anatomical structure; namely, their thin walls, more readily admits of the escape of the leucocytes by their amoeboid movements into the surrounding structures. The red corpuscles also escape, but in a very limited number. At the same time of the passing of the leucocytes, there is also the transudation of the *liquor sanguinis*, forming the serous effusion.

The escaped leucocytes not only increase by dividing and multiplying, but they also excite the tissues into which they find their way, into increased cell-proliferation. This in turn causes a greater nutritive activity, with the resultant formation of the cell-growth, which plays such an important part in the pathology of the lesion called inflammation. This new cell-growth is very unstable and it soon passes into retrogression or the breaking down stage, with the production of pus. Pus being formed it dissolves alike both the *hard* alveolar, the dental organs, and soft structures, thus completely destroying the involved tissues, and ending a cycle of changes, only again to be renewed and cause further destruction of the parts by continuity and contiguity.

This disease may extend to one or more teeth, from the adjacent structures, in one or the other jaw; or in its more aggravated forms it may involve the alveolar arch of both jaws, with the final loss of all teeth implicated. The close anatomical relation of the structures within the tooth substance and those without, will at once suggest the dangers and results liable to the pulp when the outside tissues are diseased.

In a very acute case, the root membrane undergoes a gangrenous metamorphosis with its attending results.

In the more chronic forms, along with the destruction of the alveolus, the cementum and dentine of the tooth structure are changed by the pathological process of *necrosis* and resorption.

Of course, this cycle of changes is increased or retarded by the intensity of the inflammation and the resistive powers of the individual.

*Symptoms:* The symptoms of this disease are both objective and subjective. In the milder forms or in the earlier stages of this lesion, we notice the red and congested appearance of the gums, the soft boggy look and feeling, with more or less serous effusion. If the congestion be of the passive character, the gums will be more or less purple. During the acute stages, the disease is attended with pain. In the latter or chronic stages, the gums present more of an angry look, a higher degree of redness, with a greater degree of infiltration of the surrounding soft structures, with blood, liquor sanguinis, and pus. The pus is present in such quantities in some cases as to be plainly seen around the necks of the teeth; and, if not in sight, it may be very easily brought to the surface by pressure upon the gums. In the less severe cases, the pus is only seen upon the facial segment of the gum, but it is found upon the lingual when the affection is more advanced.

The pressure of the tongue and its constant sucking action upon the lingual surfaces of the alveolar process, will undoubtedly account for the seemingly absent purulent matter upon the lingual segment in the less severe or aggravated cases.

The breath is tainted with a characteristic odor varying in intensity according to the severity of the case.

The subjective symptoms in the advanced stages of Pyorrhoea Alveolaris are a bad taste in the mouth, especially in the morning, due to the quantity of purulent matter that has been discharged into the mouth during the night. The tooth or teeth are found to yield under pressure, due to the breaking down of the proper support, and in the advanced stages the disease may be attended with neuralgic pains in the face, head, arms, and chest: and by careful examination some point or points may be found which, upon touching, may bring about the pains in one or more of the above mentioned parts. The acute sense of taste is more or less destroyed.

*Course, Duration, and Termination:* The course of Pyorrhœa Alveolaris is acute, sub-acute, and chronic and the outset is at times obscure. This lesion may be developed and run its course within a few months, or it may take years to bring the disease to its full development. The termination of this trouble is complete destruction of the tissues involved, with the dental organs as well, unless arrested in its course with remediable agents. It is not subject, only in a very slight degree, to remediable agents, in the very last stages of the disease. If we are successful in arresting the disease, we cannot restore the true anatomical relations between the hard and soft structures.

*Diagnosis:* Pyorrhœa Alveolaris is not confounded with any other lesion of the oral cavity. The trouble is easily recognized, except in its early stages, and when the disease commences in isolated spots on the alveolar arch, then it may be easily overlooked.

*Treatment:* According to the etiology of this disease, the treatment naturally divides itself into *local* and *constitutional.*

In the specific cases which are of a *strumous diathesis,* such constitutional treatment should be employed as will restore the powers of assimilation and nutrition to the proper functions. In fact, the whole system must be put into its best condition. And this is met or aided by strictly observing personal hygiene, careful diet, regular habits, and the giving internally of the elixer of calisaya, iron, and strychnine, or citrate of iron, or bark, iron, and hypo-phosphites of lime, or some one or more combined of the many forms of bark, iron, and lime. Of course, in the specific cases, we must also use *local treatment.*

In the non-specific or acquired cases, the cause being mechanical, chemical, or thermal, we must first remove the cause and then apply such antiphlogistics as shall arrest the disease, and overcome the existing stages of inflammation; and this may be accomplished in some such manner as follows: If the exciting cause be of a mechanical irritation, such as the deposits of calcarious matter upon and around the necks and roots of the teeth, it must be very gently removed with Scalers, three-edged chisels, and other necessary instruments at our disposal: and in performing the surgical operation, care must be taken lest we defeat our object in a great measure by excessive mutilation of the adjacent soft structures. We must ever remember the surgical law, that all wounds do not terminate alike in all persons. The restoration of the parts depending at times upon the peculiarities and constitution of the patient. A second point to be observed in removing the mechanical irritation is *not to be in a hurry.* Do not try to remove too much at one sitting. The quantity that can be removed without injury will depend upon the quantity of deposit, the degree of inflammation, and the condition of the patient. No absolute rule can be given for the above but we must be governed by general principles.

If chemical irritants, as mercury and fumes of phosphorus are the primary causes, we must first remove the patient from the source of contamination and then free the system of the irritants by the proper systemic treatment. We may correct the mercury by internal administration of chlorate of potassium, and the phosphorus by the aid, internally, of turpentine, iodide of potassium, and bi-chloride of mercury.

Should thermal changes be the cause, we have simply to bring about their cessation.

When catarrhal troubles are the cause, we must meet them with the proper agents, and the secondary consequence will cease.

Having eliminated the primary causes, we have now to overcome the existing inflammation, which, if found in the acute stage (and by the way we rarely get the patient when in this stage), should be treated by mild non-irritant remedies, as glycerine, aconite, and belladonna, remembering not to use astringents in any form in the acute stage. In the sub-acute we may use mild astringents as glycerine ʒj; tannin grs.v; or a solution of hydrastine and water, equal parts; or nitrate of silver, grs.ij to water ℥j.

When the chronic form is reached we have only to increase the power or strength of our anti-phlogistic remedies according to the intensity or chronicity of the disease. When the disease is attended with suppuration, then we must employ astringents, antiseptics, and disinfectants. Remove the broken down structures and provide as free drainage as possible.

In this last stage of the disease there are two drugs, iodide of zinc and peroxide of hydrogen, that fulfill the requirements for the astringent and disinfectant properties exceedingly well.

The antiseptic qualities are met by a mouth wash as follows: listerne, ℥ij; oil of eucalyptus, gtts.x, and water f℥viij; and a mouth or tooth powder as follows: prepared chalk ʒvi, bicarbonate of soda grs.x, prepared oris root ʒij, and oil of wintergreen gtts.v. The mouth wash is directed to be used one-half to one teaspoonful into a quarter of a glass of tepid water, after each meal and at bedtime. The wash must be held in the mouth from one to two minutes, and thoroughly forced in and around the teeth in order to get the desired results. The powder is used every, or every other, or every third night at bedtime, just before using the mouthwash as the case requires.

Mode of Administration of Peroxide of Hydrogen and Iodide of Zinc and their Chemical Relations: A hypodermic syringe with a blunt needle is the instrument used in injecting the agents in solution, into the diseased pockets. Here care must be used not to cause injury, for, if we do, no advance can be made. The syringe being filled *first* with peroxide of hydrogen, and refilled if needed, the needle is passed gently into each diseased pocket and around the neck of each diseased tooth beneath the margin of the gum. This solution coming in contact with the purulent matter, at once consumes the carbonaceous matter and produces, as by-products, water, nitrous acid, and carbonic acid gas. The chemical changes in the carbonaceous matter prove to be the required disinfectant, while the nitrous acid thus formed gives us a tonic or stimulating astringent.

With the pockets yet filled with the solution of peroxide of hydrogen and its formed by-products, we again, having washed out the syringe, re-fill it with a solution of iodide of zinc, two to five grains to the drachm of distilled water and again we pass the needle around and into all diseased pockets in the same manner as we did with the peroxide of hydrogen. No sooner do the two solutions come in contact, than a third medicinal property is formed by the liberating of the *iodine* from the zinc, and the forming of an aqueons solution of iodine, which is a non-irritant, and has the therapeutical properties of causing inflammatory products to be absorbed.

The applications are made every day, morning and evening, for one or two weeks, and as the disease disappears we lessen our number of treatments.

When we desire to remove, by slough, any part of the diseased structures and bring about a speedy resolution, it may be effectually accomplished by an application of Robinson's remedy, or equal parts of iodine crystals and creosote.

*Objection:* There is one objection to the above treatment and that is its administration is a trifle inelegant; but at the suggestion of my friend Dr. W. C. Ames, of Chicago, we are now able to produce free iodine at just the point where we desire it by the electrolysis of iodide of potassium. With the foregoing treatment, with more or less modifications, we can cure the disease known as Pyrrhoea Alveolaris, but we cannot restore the lost parts.

Owing to the length of this paper I will cite just two typical cases taken from my record of cases on Pyorrhœa Alveolaris and treated in accordance with the above remarks.

Case No. 1. Mrs. M. aet. 45; both dental arches complete; not much deposit upon the teeth; bad taste in the mouth: breath very offensive; all teeth in mouth loose and sore; gums highly congested and swollen, and so thoroughly infiltrated with pus as to well up around the cervical border of each tooth. The probe could be passed along the side of the roots of several teeth to nearly their apex. She had noticed some trouble with her gums for seven years past. For the last two years she had been troubled with severe neuralgic pains in the head, breast, shoulder, and down the arm to the elbow. I could bring on the pain to the right elbow at once by touching the buccal side of the anterior root of the right lower, first molar. She had sought for relief for the past three or four years, but was told that the extracting of her teeth was the only remedy. She did not want to lose her teeth, hence she endured the pain.

Her directions with regard to personal care were about the same as pointed out above. Her teeth were cleansed and the mechanical irritations of deposits removed, which was the only assignable cause that I could give; although her health at that time was not just as good as it might have been. General systemic tonics were ordered to be taken after each meal, and we made applications of peroxide of hydrogen and iodide of zinc, night and morning for two weeks with marked improvement; no pain, no pus, and little motion to teeth. At this point she was called away and I provided her with a solution of iodide of zinc, four grains to the drachm, and directed her to apply it to the gums once a day with a small camel's hair brush, which she did for one year, at the end of which time I saw her again and found the disease somewhat improved, besides arresting its onward march. I again advised continuance of the same treatment.

Case No. 2. Mr. H. aet. 25; both dental arches complete; no deposit upon the teeth; severe burning pains at necks of six, lower, anterior teeth; gums red but not swollen; by pressure upon gums a light saneous fluid would appear at the necks. Trouble had existed for four weeks. General health excellent. At that time no cause could be assigned. He was given the above described mouth wash and tooth powder, and we applied the peroxide of hydrogen and iodide of zinc treatment once a day for five days with complete success.

Two weeks elapsed and he again presented himself with the same symptoms, only in a different place; namely, the left lower *bicuspids* and *first molar*. A few treatments and the trouble was at an end. Something like another week of time went by and he again came to see me; this time complaining of the upper central incisors and upper left bicuspids. We easily corrected the trouble, but the difficulty was to find out the cause, which, at last, by a happy hit, proved to be the smoking of cigarettes. This thermal change being removed the cure was complete.

